# The incidence and intensity of postoperative pain and Flare-up following the use of three different intracanal medicaments in teeth with posttreatment apical periodontitis: a randomized clinical trial

**DOI:** 10.1007/s00784-024-05760-w

**Published:** 2024-06-08

**Authors:** Adile Esen Angın, Hicran Dönmez Özkan, İlkim Pınar Saral, Berdan Aydın

**Affiliations:** 1 Department of Endodontics, Faculty of Dentistry, Aydın Adnan Menderes University, Aydın, 09100 Turkey; 2 Department of Endodontics, Faculty of Dentistry, Dokuz Eylül University, İzmir, Turkey

**Keywords:** Calcium hydroxide, Chlorhexidine gel, Endodontic postoperative pain, Flare-up, Intracanal medicament

## Abstract

**Objectives:**

This randomized clinical trial aimed to compare the effect of intracanal medicaments on the incidence of postoperative pain and flare-up with posttreatment apical periodontitis (PTAP) of retreatment cases.

**Materials and methods:**

One hundred twenty patients diagnosed with PTAP with single-rooted teeth with single-canal without spontaneous pain or swellings were included and randomly divided into three groups according to the intracanal medicament used. Intracanal medicaments were placed into the root canals following the removal of previous root canal fillings and re-instrumentation. Calcium hydroxide (Ca (OH)_2_), chlorhexidine gel (CHX), calcium hydroxide and chlorhexidine gel combinations were used as intracanal medicaments. Postoperative pain scores were recorded at 6 and 12 h and 1, 2, 3, 4, 5, 6, and 7 days using a visual analog scale (VAS). Sensitivity on percussion, spontaneous pain, swelling, antibiotic and analgesic requirements of the patients were evaluated during clinical examinations performed postoperatively after 2 and 7 days.

**Results:**

There were no statistically significant differences between groups in terms of VAS scores following the intracanal medicament application (*p* > 0.05). However, compared to the patients of 20–34 and 50–65 age groups, greater VAS scores were observed in patients of 35–49 age groups at 12 h, and 3, 4, 7 days (*p* < 0.05). Flare-up was observed in only one patient in the CHX gel group, and no flare-up was observed in other groups.

**Conclusions:**

Similar postoperative pain incidence in all experimental groups indicates that all three medicaments are clinically acceptable in inter-appointment management of retreatment cases in terms of post-endodontic pain and flare-up.

**Clinical relevance:**

In this randomized clinical trial, three different intracanal medicaments were utilized in nonsurgical endodontic retreatment and their effect on postoperative pain and flare-up incidence was examined. Thus, this study will be a significant contribution in the decision-making during clinical practice; since there are a limited number of prospective clinical trials in the literature about the severity of pain following retreatment procedures including intracanal medicament use.

## Introduction

Postoperative pain and flare-up are among the clinical complications encountered during and after endodontic treatment [1, 2]. Although endodontic treatment done using contemporary techniques provides appropriate biological results, preventing pain during and after the treatment is as much desired [3]. Postoperative pain occurs as a result of many factors such as the presence of pain preoperatively and procedural variations. Factors such as gender and age, presence of a systemic disease, condition of the pulp, presence of preoperative pain, number of appointments, use of intra-canal medications and localization of the tooth in the arch may stimulate postoperative pain and flare-up [4]. Many studies have reported that the frequency of flare-up is higher in retreatment cases compared to the flare-up rate of patients with primary root canal treatment [4–7]. One of the most important causes of postoperative pain is microorganisms that cannot be eliminated from the root canal. Microbial flora in cases with unsuccessful endodontic treatment history is different from that of primary root canal infections. *Enterococcus faecalis* constitutes a small part of the flora in cases where root canal treatment is performed for the first time, while plays a major role in the etiology of persistent periradicular infections with root canal treatment [8]. Therefore, in retreatment procedures, although microbial eradication is mainly achieved by chemomechanical preparation, root canal medicaments may help to eradicate microosganism from the root canal [9, 10].

Calcium hydroxide has been the most used root canal dressing in endodontic practice since the early 1930s [11]. Although it is the most preferred medication, it does not show an equal effect against all the bacterial species in the root canal system [12, 13].

Chlorhexidine, which has been widely used in dentistry since the 1970s, was recommended in irrigation and as an intra-canal disinfectant in endodontics. In a study, its solutions, gels, and controlled release preparations were found effective against resistant microorganisms such as *Enterococcus faecalis* and *Candida albicans* [14]. Owing to its viscosity, chlorhexidine gel was found better in mechanical cleaning compared to its liquid counterpart [15]. A study reported that when used as an intra-canal disinfectant, chlorhexidine is more effective than calcium hydroxide against *E. faecalis* [16]. However, chlorhexidine alone cannot form a physical barrier and cannot provide radiopacity [12]; thus, its use combined with calcium hydroxide may offer such features.

The synergistic effect of chlorhexidine and calcium hydroxide mixture increases the antimicrobial activity of calcium hydroxide while preserving its barrier function [12, 17–19]. There is limited knowledge about the effectiveness of this combination on postoperative pain and flare-up incidence in retreatment cases.

Therefore, the aim of this study is to evaluate the effect of chlorhexidine, calcium hydroxide, chlorhexidine and calcium hydroxide mixture as intra-canal medicaments on postoperative pain and flare-up incidence and the frequency of analgesic use in teeth with PTAP. The null hypothesis tested in this study is that the type of intra-canal medicaments used would not affect the incidence and the intensity of post-treatment endodontic pain and flare-up incidence.

## Materials and methods

The research protocol was recorded in the www.ClinicalTrials.gov database (National Library of Medicine 8600 Rockville Pike, Bethesda, MD 20,894, United States) under ID NCT05052814 and the study was approved by our university’s Clinical Research Ethics Committee (Protocol ADUDHF2019/063) and Pharmaceuticals and Medical Devices Agency of our country (Protocol 2019/ 079).

The minimal estimated sample size for each group was computed as 34 based on previous research data [20] [alpha-type error of 0.05, the power of the study was chosen as 0.95 and calculated by G*power V.3.1.9.2 software (Heinrich Heine, University of Düsseldorf, Düsseldorf, Germany)]. To increase the statistical power and consider the potential patient dropouts, 40 patients per group were enrolled. Thus, a total of 114 patients were included in this study.

### Patient selection and eligibility criteria

The patients were selected from those referred to the Hospital of Aydın Adnan Menderes University of Dental Faculty from March 2019 to August 2020. All patients meeting the inclusion criteria were invited to participate in the study. One hundred and twenty systematically healthy patients aged between 20 and 65 years met the criteria and agreed to participate in the study. Patients who had root canal-treated single-rooted incisor or mandibular premolar teeth with a single root canal at least or equal three years ago but still had PTAP were included in this study. Failure of the previous root canal treatment was determined by clinical and radiographic examinations. Teeth with clinal signs and symptoms for the requirement of retreatment except for cases with preoperative swelling, spontaneous pain, severe percussion and palpation were included in this study.

All inclusion and exclusion criteria are listed in Table 1. All participants were first informed about the study design and clinical treatment procedures with risks and signed a written informed consent form.

**Table 1:** Inclusion and exclusion criteria in the present study

Inclusion criteria	Exclusion criteria
Patients between 20 - 65 years of ages	Pregnant patients and patients in lactation period
Patients who agree to participate this study	Having used corticosteroids in the last 6 months
Patients had not used analgesic or antibiotics in last 7 days	Individuals with systemic diseases (endocarditis, immune system diseases, etc.) requiring antibiotic prophylaxis
Single root and single-canal incisor, canine, premolar teeth that have been endodontically treated only once	Having received immunosuppressive therapy within the last week
Asymptomatic teeth with previous endodontic treatment but with abscess experience or pain on chewing/percussion history developed within last 12 months.	Patients who had systemic or allergic sensitivty for the NSAIDs and local ananesthetics
Teeth, although asymtomatic, but with newly developed periapical radiolucency within two years compared to the radiographic findings present 2 years ago at the time of root canal tretament was done.	The presence of advanced periodontal disease (probing depth > 4 mm)
Teeth with periapical radiolucency, but with increased or unchanged according to pre-treatment radiography	The presence of a foreign body in the root canal that prevents entry (broken file, post, etc.)
Patients with good oral hygiene	Fracture or crack in the root
	Teeth that cannot reach the working length due to calcification in the root canal and step formation
	Teeth that cannot be restored due to excessive loss of material in the coronal structure
	The presence of more than one adjacent tooth requiring endodontic treatment that may cause reflected pain in the same patient.
	Teeth that develop any complications (breakage of endodontic file, perforation, inability to determine the working length with the apex finder) during the removal of the canal filling material.

### Random sequence generation and allocation concealment

The volunteers were randomly assigned to three different medicament groups. The allocation was performed according to Consolidated Standards of Reporting Trials 2010. Stratified randomization was performed for each group according to gender and age. After patients were divided based on gender and age groups, they were randomly placed in the medicament groups to make an equal distribution of the type of medicament used. To implement a random placement of patients, different blocks were designed with the combination of covariates; and a simple randomization procedure, envelope selection, was applied within each block to assign participants to one of the blocks.

### Treatment protocol

All root canal treatments were performed by the same clinician. During the diagnostic examination, periapical radiographs were obtained using a phosphor plate (Vistascan Mini Easy, Dürr Dental) and using the long-cone paralleling technique under standard exposure conditions and recorded. After the clinical and radiographic evaluations of relevant teeth; the findings including spontaneous pain, swelling, fistula, restoration type and condition, caries and fractures were recorded on the case report forms. Tests such as percussion, palpation, mobility and periodontal probing were performed to determine the presence of preoperative pain. All patients received 2-visit root canal retreatments.

After administration of local anesthesia (2 mL, 4% articaine hydrochloride containing 1:100 000 adrenalin), a rubber dam was applied for the isolation. No additional local anesthesia was given since patient comfort was provided with the delivered amount. Following the preparation of the access cavity, ProTaper Universal Retreatment files (Dentsply Maillefer, Ballaigues, Switzerland) were used at 500 rpm and 3 Ncm torque to remove the root canal filling. D1 file (30 / 0.09) was used in the coronal third, D2 file (25 / 0.08) in the middle third, and D3 file (20 / 0.07) in the apical third region. The working length was determined to be 1 mm shorter than the value (0.0) indicated by the electronic apex locator (Raypex 6, VDW, Munich, Germany) and confirmed radiographically. Apical patency was established with a size 10 K-file. Next, the shaping procedure was completed at the working length using ProTaper Next rotary files: X1, X2 and X3 (Dentsply Maillefer, Ballaigues, Switzerland), respectively. In large canals where the use of X3 files results in under-instrumentation of apex, shaping was completed with X4 and X5 files. No chemical solvent was used to remove the previous root canal fillings. A 27-gauge notched type irrigation needle (Endo Eze; Ultradent Products Inc. South Jordan, USA) was placed loosely 2 mm shorter of working length while performing 2 mL, 5.25% NaOCl irrigation between each file. After the last used file, shaping for retreatment was considered complete when there was no residual canal filling observed under 2.5x magnification and the irrigation solution was clear from debris. Then, a periapical radiograph was taken to verify the complete removal of the filing materials. If any remaining material is visible on the radiograph, shaping was completed with X4 and X5 files and a size # 50 Hedström file was used until complete removal of the previous filling was achieved. After that, final irrigation was done under activation (Endoactivator, Dentsply, Tulsa, USA) using 2 mL of 17% EDTA followed by 4 mL of 5.25% NaOCl. The endoactivator was run for 20 s between 1 mL 5.25% NaOCl irrigation. The root canal was rinsed with sterile distilled water and dried with sterile paper points. Finally, to minimize technical variations in medicament placement which could affect postoperative pain, all medicaments applied with a lentulo spiral was used (Dentsply Maillefer, Switzerland) 2 mm minus the root canal length as follows:

#### Group 1

Calcium hydroxide paste (Ultracal XS; Ultradent South Jordan, USA).

#### Group 2

2% Chlorhexidine gel (Gluko-Chex, Cerkamed, Stalowa Wola, Poland).

#### Group 3

Equal amounts of calcium hydroxide (Ultracal XS; Ultradent South Jordan, USA) and chlorhexidine gel (Gluco-Chex, Cerkamed, Stalowa Wola, Poland) were placed on a sterile mixing pad. A sterile spatula was then used to stir until a homogeneous mixture was obtained.

After the visual observation of the canals filled with medicament, sterile teflon tape was placed in the canal orifice and the access cavity was sealed with glass ionomer cement (Kavitan ™ Plus; Pentron, SpofaDental, Czech Republic).

### Postoperative pain evaluation

Postoperative pain levels were evaluated for seven days using a VAS scale. Postoperative pain scores were recorded at 6, 12 hours and at 1, 2, 3, 4, 5, 6 and 7 days after the medicaments were placed. In the VAS scale given to the patient two opposite limits of the parameter are marked on both ends of a line prepared as 0-100 mm. According to the scale, while ‘no pain’ is marked with zero, ‘unbearable pain’ is marked with 100 on the line [21]. The patients were asked to evaluate their own pain status by marking the line on the specified days and times. Patients who could not communicate to submit the evaluation forms were excluded from the study. The patients were prescribed 400 mg ibuprofen (Brufen, Abbott, IL, USA) and instructed to use it only for severe pain. Frequency and time of use of analgesics were recorded. In addition to the documentation, oral examinations were performed on the 48th hour and at 7 days. Sensitivity on percussion, spontaneous pain, swelling of surrounding tissues and antibiotic requirement were also examined and recorded.

### Evaluation of previous root canal filling level

Evaluation of the status of primary root canal fillings was performed by two independent endodontists, who were not included in the study and had at least three years of experience, clinically and on the periapical radiographs. Canal fillings terminating at the radiological apex and 2 mm within are ‘acceptable’; canal fillings shorter than 2 mm of apex are ‘short’ and gutta-percha seen beyond the radiological apex are considered ‘overfilled’ [21, 22].

### Statistical analysis

IBM SPSS Version 25 package program (IBM © Corp., Armonk, NY, USA) was used for statistical analysis of data. The normality of distribution was evaluated by using Lilliefor’s corrected Kolmogorov-Smirnov test, kurtosis-skewness plots and histograms. In statistical analysis, Pearson’s chi-square test was used for categorical data. The exact chi-square test was used if the value observed in the multi-level chi-square test was more than 20% and less than 5%, using the standard residual method in post hoc analysis.

The non-parametric Kruskal–Wallis H test was performed to compare numerical variables (age, VAS scores) amongst the three groups and the SPSS pairwise comparison module for the post hoc evaluation. The Mann-Whitney U test was used for comparisons of two independent groups. The Friedman test was used to evaluate the changes in pain scores over time. Categorical variables (gender, analgesic intake) were compared amongst the groups using the chi-squared tests. Spearman correlation analysis was used because parametric assumptions were not met in comparisons of two different measurement data. The consistency between the observer evaluations was evaluated with Cohen’s Kappa test. There is no missing data in the data set; the type I error level was set at 0.05.

## Results

**Fig. 1:**
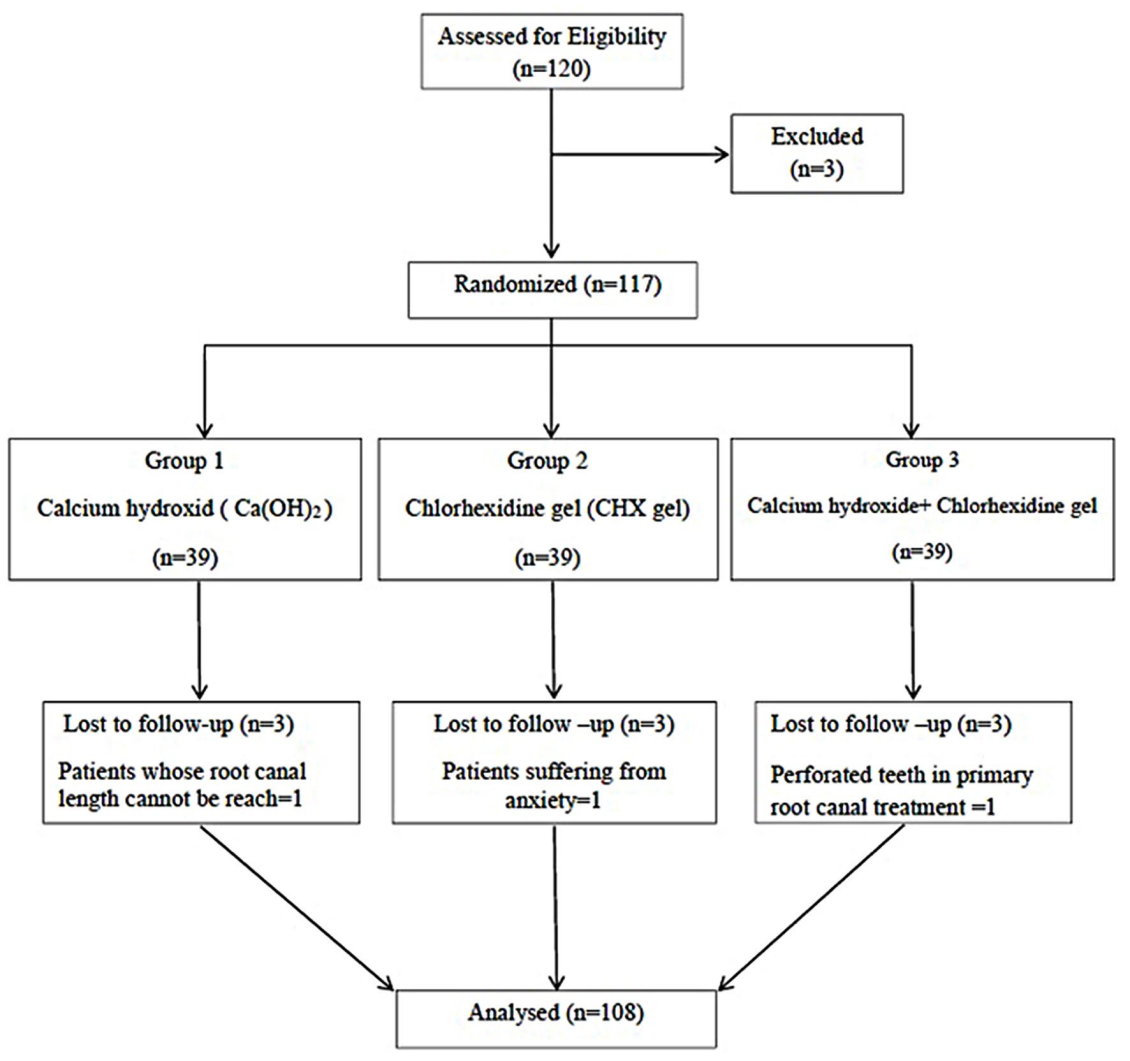
Flow diagram of the participants

Three patients declared their leave from the study after the first visit. Additionally, a total of nine patients, three per group were excluded from the study due to various reasons such as apical restriction, anxiety, and perforations due to the previous root canal treatment. Thus, they were excluded from the final analysis, which included a total of 108 retreatment cases performed on 108 patients (Fig. 1). Forty-one of the treated teeth were maxillary incisors, 26 of the treated teeth were maxillary premolars, 6 were mandibular incisors and 35 were mandibular premolars. All the root canals have a single root canal. There were no statistically significant differences between the distribution of the types of teeth in medicament groups. (exact chi-square test, *p* > 0.05, Table 2)

**Table 2:** Distribution of the types of teeth from the medicament groups

	Treated teeth, n(%)				*Chi-Square*			
	Maxillary Incisior	Maxillary premolar	Mandibular Incisior	Mandibular Premolar	Total	X^2^	df	p
Ca(OH)2	14 (38.89%)	11 (30.56%)	1 (2.78%)	10 (27.78%)	36 (100%)	5.589	6	0.487
CHX JEL	17 (47.22%)	5 (13.89%)	2 (5.56%)	12 (33.33%)	36 (100%)			
Ca(OH)2 + CHX JEL	10 (27.78%)	10 (27.78%)	3 (8.33%)	13 (36.11%)	36 (100%)			
Total	41 (37.96%)	26 (24.07%)	6 (5.56%)	35 (32.41%)	108 (100%)			

18 male (50%) and 18 female (50%) patients between the ages of 20–65 were included per group. Patient age groups were divided into three age groups: 20–34, 35–49 and 50–65; and a stratified randomization was performed. When the correlation between age and postoperative pain is evaluated; at 12 h, patients between the ages of 35–49 had significantly greater pain compared to the patients of 20–34 (*p* = 0.033) and 50–65 age range (*p* = 0.017). There was no statistically significant difference at 3, 4 and 7 days except for patients of 35–49 age group who had significantly more pain compared to patients between the ages of 20–34 (*p* < 0.05).

No significant differences were observed in any of the following parameters: gender (exact chi-square test, *p* > 0.05), presence of a periapical lesion (exact chi-square test, *p* > 0.05), quality of obturation (exact chi-square test, *p* > 0.05), type of the coronal restoration (exact chi-square test, *p* > 0.05), location of the teeth (exact chi-square test, *p* > 0.05) and presence of fistula (exact chi-square test, *p* > 0.05).

There were no significant differences amongst the tested medicament groups at any of the assessed time intervals based on VAS scores (*p* > 0.05). Additionally, no statistically significant difference was observed in the tested medicament groups when a periapical lesion was present (Mann-Whitney U test, *p* > 0.05); while patients with no fistula had significantly greater pain values after 24 h compared to patients with fistula (Pearson chi-square test, *p* > 0.05; Mann-Whitney U test, *p* < 0.05).

When correlation is tested between the presence of coronal restoration and pain, only patients with coronal restoration had more pain postoperatively at 6 h (Pearson chi-square test and Mann-Whitney U test, *p* < 0.05).

No significant difference was found between the apical extension of the previous canal fillings in different medicament groups (Exact chi-square test, x^2^ = 3.836, df = 4, *p* > 0.05). When the correlation between the apical extension of the primary root canal fillings and pain was evaluated (Kruskal-Wallis H test); significantly greater pain was observed in the short and overextended groups at 24 and 48 h compared to the cases with acceptable root canal filling levels (*p* < 0.05).

There was no statistically significant difference between the groups in terms of percussion, spontaneous pain and swelling when the level of postoperative pain was compared at 48 h and 7 days of clinical examinations (*p* > 0.05).

**Fig. 2:**
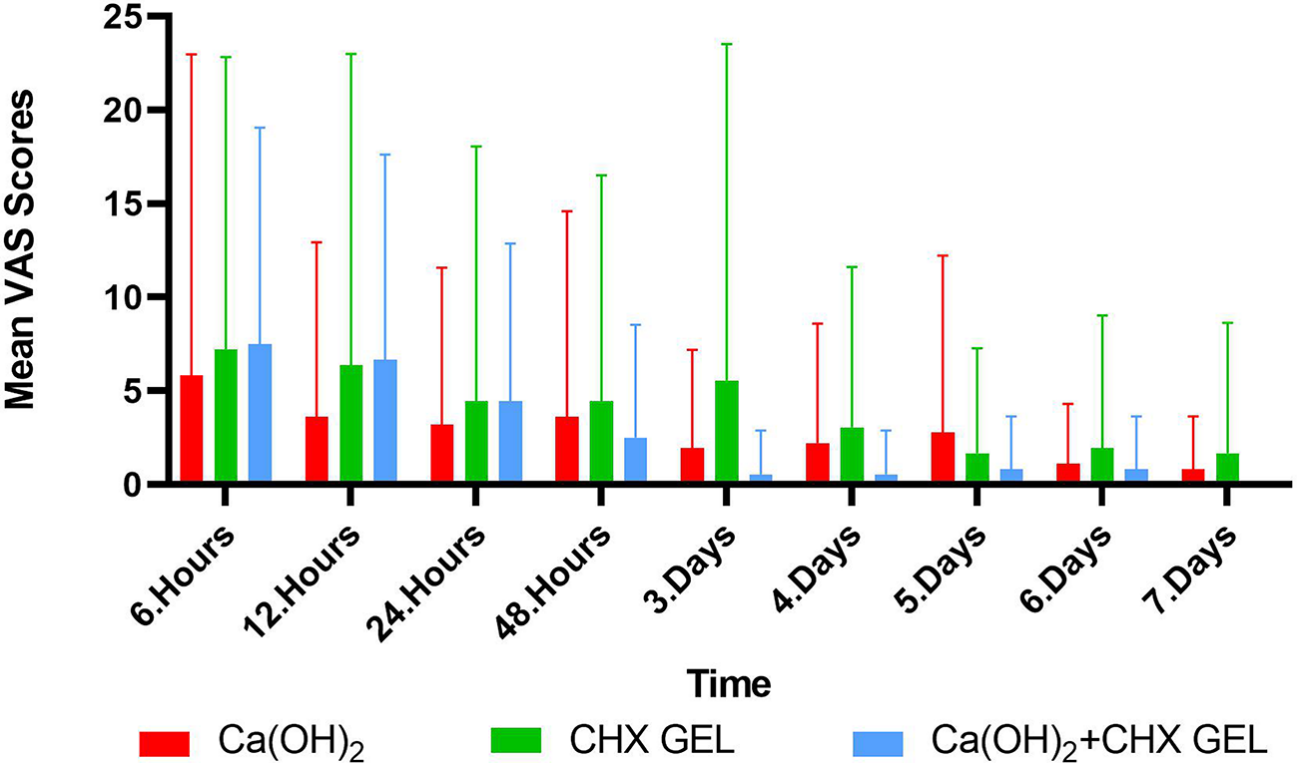
Boxplot chart shows mean pain values and reduction in pain over time

The distribution of pain levels experienced by patients on different medications during follow-up periods is shown in Fig. 2. While the Ca(OH)_2_ group had the lowest VAS scores postoperatively for the first 24 h compared to the other medicament groups. After the first 24 h, Ca(OH)_2_ group VAS scores reflect a fluctuating postoperative pain until day 7 (scores were increased after 24 h, started to decrease at the 48th hour, but started to increase again after the 3rd day. The increase was continued until the 5th day and then a decrease occurred). While the Ca(OH)2 + CHX gel group had a high VAS score at the first 12 h, it showed a continuous declining trend with the lowest VAS score after 48 h compared to the other groups. The VAS score of the CHX gel group was greater compared to the scores of the other groups.

The Kruskal-Wallis H test was used to evaluate whether there was a difference between the postoperative pain values between the groups at the 6, 12, 24, 48 h and at 3, 4, 5, 6, and 7 days after the medicament placement. When all the time pain levels observed were evaluated, no statistically significant difference was found between the groups in terms of VAS scores (*p* > 0.05).

When the frequency of drug use was investigated a statistically significant difference was found between groups (Exact chi-square test, *p* < 0.05). According to the results of Spearman correlation analysis conducted to analyze whether there is a correlation between frequency of drug use and VAS scores, a positive moderate statistically significant correlation was found (Spearman’s *r* = 0.424, *p* < 0.001) indicating that VAS score increases while also drug use increases.

## Discussion

Postoperative pain following root canal treatment is mostly associated with microorganisms remaining in the root canal system. Eradication of microorganisms is rather harder in retreatment cases due to the presence of remnants of previous root canal filling. As a result, the frequency of flare-up was found higher in retreatment cases compared to the flare-up rate of patients with primary root canal treatment [4–7]. Thus, using medicaments after a careful chemomechanical preparation may improve disinfection of the root canal system in retreatment cases [9, 10]. There are limited number of clinical trials examining the effect of intracanal medicaments on postoperative pain and flare-up incidence in nonsurgical endodontic retreatment. Thus, the present study aimed to evaluate the effect of the three different intracanal medicaments on postoperative pain and flare-up incidence in nonsurgical endodontic retreatment. Based on the findings of this study, there were no significant differences among the tested intracanal medicaments; so, the first null hypothesis could not be rejected.

If the root canal fillings are exposed to an oral environment, contamination with saliva takes place. Saliva contamination leads to bacterial growth and penetration throughout the entire canal [4]. If root canals are left open for a long time some bacteria decrease in number or even disappear while the remaining ones may turn into adaptive phenotypes that resist treatment [23]. Postoperative pain and flare-up may develop due to changes in periapical tissue pressure and microbial factors after retreatment [24]. Therefore, in studies evaluating the severity of postoperative pain in nonsurgical endodontic retreatment, the presence and quality of coronal restoration before treatment should be examined. Teeth with marginal defects in coronal restoration demonstrate greater postoperative pain after root canal treatment [23]. Contrarily to the previous findings, the present study represented patients with an intact initial coronal restoration had statistically more pain after 6 h postoperatively compared to the patients whose coronal restoration was absent in the first appointment. A selective process occurs over time that allows anaerobic bacteria to predominate if the cavity is sealed after root canals become infected with indigenous oral bacteria [25–27]. When pain and the type of bacteria present in root canals are correlated; it was shown that root canals from symptomatic teeth harbored more obligate anaerobes and a bigger number of bacterial species than the asymptomatic teeth [28].

A relationship between systemic diseases such as uncontrolled diabetes, allergic patients, corticosteroid use, and postoperative pain was shown [4, 24] while a study [9] reported no relationship between them. Thus, only healthy patients with no systemic diseases were included in the present study. Also, patients who had not used any analgesic, anti-inflammatory or antibiotics within one week prior to the first appointment were chosen; since the long-term effects of antibiotics, analgesics and anti-inflammatory drugs change the perception of postoperative pain.

Patients with preoperative pain have a greater potential of developing pain and flare-ups after treatment [1, 4, 9]. Therefore, cases with pain and swelling were not included in the present study.

Postoperative pain was recorded as the greatest within 48 h after treatment [7, 23, 29, 30] and the follow-up was usually done for a week [23, 30, 31]. Similarly, the same follow-up methodology was adapted in the present study.

The present study considered a stratified randomization design, demonstrating a more reliable correlation between demographic properties and the incidence of postoperative pain, and flare-up when compared to the previous studies. Similar to the previous studies [9, 23, 31, 32] gender of individuals showed no significant correlation with postoperative pain and flare-up recorded in medicament groups.

Torabinejad et al. [4] reported that postoperative pain increased in patients of 40–59 years of age, while postoperative pain decreased in patients younger than 20 years. Jabeen and Khurshiduzzaman [33] declared that older patients felt more postoperative pain than younger patients. Researchers stated the reason for less postoperative pain in the younger group could be a lack of dental experience and physiologic tolerance to pain. Likewise, in the present study, compared to the patients of 20–34 age group; significantly greater pain was recorded in patients of 35–49 age group postoperatively 12 h, 3, 4, and 7 days.

The pressure created by the periapical abscess is drained by the fistula; thus, if present, a fistula may prevent sudden pain increase and swelling. The risk of acute exacerbation was reported to be small in the presence of a fistula [4]. Similarly, in the present study, patients with no fistula had significantly greater pain at 24 h postoperatively.

Postoperative pain decreases as the periapical lesion volume sizes increase [29]. This phenomenon might be explained by the presence of sufficient space for pressure distribution in cases with large periapical lesions [34]. However, flare-ups develop less frequently in teeth without apical periodontitis [5]. On the other hand, as the size of the periapical lesion increases, the frequency of flare-up development increases [35]. In this study, no statistically significant relationship was detected between the presence of periapical lesions and postoperative pain. Small sample size of studied patients (*n* = 108) in our study compared to the large sample size of previously published studies may explain why a correlation cannot be found between periapical lesions and postoperative pain [6, 36].

When postoperative pain was evaluated after retreatment was completed [37]; only teeth with root canal filling 2–4 mm shorter than the apex were studied to eliminate the detrimental effect of overextending previous root canal filling on periapical tissues. When the effect of primary root filling levels on postoperative pain after retreatment attempt were evaluated in a recent study, within 24 h greater pain was experienced by the patients with short initial root canal fillings [38]. In the present study, primary root canal treatment is defined as “acceptable” if it ends 2 mm shorter or within 2 mm of the radiological apex; “short” if it is positioned more than 2 mm coronally from the radiological apex; and “overextending” if it is beyond the radiological apex. According to our research results, the severity of postoperative pain was significantly greater in the cases labeled as short and overextending after 24 and 48 h postoperatively.

It has been reported that the chlorhexidine gel provides 100% inhibition of microorganisms at a depth of 200 μm in the dentinal tubules, reaching a depth of 400 μm from the 1st day and thus showing high spreadability [29, 39]. When the effect of intra-canal medicaments on postoperative pain was evaluated in a study [19] 2% chlorhexidine gel and calcium hydroxide + chlorhexidine gel group were more effective in reducing pain than the calcium hydroxide group. Rapid and sustained antimicrobial effects of chlorhexidine due to its high diffusivity were pronounced in controlling postoperative pain. Reducing or eliminating lipopolysaccharides associated with clinical symptoms such as spontaneous pain, percussion, and palpation was presented as a part of its pain-controlling mechanism [19]. However, when 2% chlorhexidine is used in irrigation it was reported that 47% lipopolysaccharides were detoxified but it is insufficient to fully inactivate them [40]. When its gel form was placed as a root canal medicament flare-up developed in four cases in the chlorhexidine (0.12%) gel group, whereas no flare-up was observed in the group with calcium hydroxide/camphorated paramonochlorophenol/glycerin paste [41]. However, no statistically significant differences were found. Similarly, in this study, although there were no statistically significant differences between the groups in terms of postoperative pain values, the pain values ​​of the CHX gel group were always the greatest. Flare-up was recorded in the chlorhexidine gel group, but only in a single case.

A combination of calcium hydroxide and chlorhexidine has a synergistic effect on lipopolysaccharides (endotoxins) produced by gram-negative bacteria. Due to the high pH (12.8) of the mixture, the ionization capacity of the chlorhexidine compound increases [16], while the contact angle of Ca(OH)_2_ decreases and thus the wettability of the root canal with medicament increases [25]. As a result, the antimicrobial effects of both compounds increased. In a clinical study, postoperative pain was significantly reduced in a 2-visit endodontic retreatment design in which Ca(OH)_2_ and 0.2% CHX gel mixture was used as an intra-canal medication, compared to the 1-visit treatments [24]. When the effect of intra-canal medicaments on postoperative pain was evaluated [19], Ca(OH)_2_ and 2% CHX mixture was found to be the most effective medication in reducing postoperative pain, followed by the chlorhexidine gel group. The least effective medicament in reducing postoperative pain was Ca(OH)_2_. Similarly, in our study, although there were no statistically significant differences in postoperative pain values ​​between the medicament groups; the Ca(OH)_2_ + CHX gel group had a high VAS score at the beginning. However, the Ca(OH)_2_ + CHX gel combination showed the lowest score after 48 h and after one week; since this combination might possess a synergistic effect against liposaccharides and endotoxins produced by gram-negative bacteria.

Taken together, conditions such as flare-up and the lack of a split-mouth design might be considered the limitations of the present study. Although, different genders and age groups were grouped and compared by using stratified randomization in our study, future studies minimizing inter-personal differences would provide more consistent results regarding pain sensation after root canal treatment appointments.

## Conclusions

Postoperative pain and flare-ups do not seem to differ when calcium hydroxide, chlorhexidine or their mixture is used as an intracanal medicament. A similar degree of pain relief indicates that calcium hydroxide or chlorhexidine-based medicaments are clinically preferable in retreatment cases to limit postendodontic pain ​​and flare-up incidence.

A greater postoperative pain is associated with elderly patients, patients without fistula and patients with coronal restoration present at time of endodontic access cavity preparation. In addition, patients with short and overextending previous root canal fillings experienced greater postoperative pain when compared to the patients with acceptable root canal filling levels. We believe greater number of patients with greater number of evaluation parameters should be considered by new clinical studies helping us to control postoperative pain and flare-up.

## Data Availability

No datasets were generated or analysed during the current study.
